# Host insulin resistance caused by *Porphyromonas gingivalis*-review of recent progresses

**DOI:** 10.3389/fcimb.2023.1209381

**Published:** 2023-07-13

**Authors:** Shuxian Jia, Xiaobing Li, Qin Du

**Affiliations:** ^1^ State Key Laboratory of Oral Diseases & National Clinical Research Center for Oral Diseases, West China School of Stomatology, Sichuan University, Chengdu, Sichuan, China; ^2^ Department of Pediatric Dentistry, West China Hospital of Stomatology, Sichuan University, Chengdu, Sichuan, China; ^3^ Department of Stomatology, Sichuan Provincial People’s Hospital, University of Electronic Science and Technology of China, Chengdu, China

**Keywords:** *Porphyromonas gingivalis*, insulin resistance, diabetes, Alzheimer’s disease, FFA

## Abstract

*Porphyromonas gingivalis (P. gingivalis)* is a Gram-negative oral anaerobic bacterium that plays a key role in the pathogenesis of periodontitis. *P. gingivalis* expresses a variety of virulence factors that disrupt innate and adaptive immunity, allowing *P. gingivalis* to survive and multiply in the host and destroy periodontal tissue. In addition to periodontal disease, *P.gingivalis* is also associated with systemic diseases, of which insulin resistance is an important pathological basis. *P. gingivalis* causes a systemic inflammatory response, disrupts insulin signaling pathways, induces pancreatic β-cell hypofunction and reduced numbers, and causes decreased insulin sensitivity leading to insulin resistance (IR). In this paper, we systematically review the studies on the mechanism of insulin resistance induced by *P. gingivalis*, discuss the association between *P. gingivalis* and systemic diseases based on insulin resistance, and finally propose relevant therapeutic approaches. Overall, through a systematic review of the mechanisms related to systemic diseases caused by *P. gingivalis* through insulin resistance, we hope to provide new insights for future basic research and clinical interventions for related systemic diseases.

## 1 Introduction

*P. gingivalis* is a Gram-negative (G-) anaerobic bacterium that is an important component of the subgingival plaque biofilm and one of the red complexes that are thought to be the main pathogen causing chronic inflammation in periodontal disease (Kuboniwa et al., 2009; Coutinho Almeida-da-Silva et al., 2019; Asthana and Chhina, 2021; Lv et al., 2021). Periodontitis is a chronic inflammatory disease characterized by the pathology of the destruction of periodontal supporting tissues, including periodontal pockets, gums, and alveolar bone. As one of the most common oral diseases, periodontitis endangers the oral health of 70% of the world’s population. a survey by Eke, Paul et al. confirmed the high prevalence of periodontitis in adults over 30 years of age in the United States, with almost 50% of the population affected (Sutton et al., 2017). If periodontitis is left untreated, pockets can form between the gums and teeth, and the spread of inflammation can cause other complications. Periodontitis is a risk factor for diseases such as diabetes, atherosclerosis, Alzheimer’s disease, aspiration pneumonia and rheumatoid arthritis, and tumors (Buduneli et al., 2014; Chacon Arboleda et al., 2021).

*Pseudomonas gingivalis* has multiple virulence factors that may be associated with the severity of disease following mixed infections. *P. gingivalis* has multiple virulence factors, including envelope proteins, gingival proteases, bacterial hairs, hemagglutinin, lipopolysaccharide (LPS), hemolysin, iron uptake transport proteins, toxic outer membrane vesicles/vesicles, and DNA (Brunner et al., 2010; Singh et al., 2011; Gao et al., 2012; Ikai et al., 2015; Bozkurt et al., 2017; Nagano et al., 2017; Bregaint et al., 2022; Kim et al., 2022; Swarup et al., 2022). These virulence factors disrupt innate and adaptive immunity, allowing *Porphyromonas gingivalis* to survive and multiply in the host, causing an inflammatory response, inducing insulin resistance, and promoting the development of systemic disease (Zhao et al., 2007). Current research evidence suggests that elevated circulating inflammatory factors induce insulin resistance and that *P. gingivalis* is associated with systemic disease, of which insulin resistance has been considered to be one of the important pathological mechanisms. Insulin is an important endocrine hormone, mainly produced by pancreatic beta cells, that regulates blood glucose levels throughout the body (Kheirollahzadeh et al., 2022). The main signaling pathways through which insulin acts include insulin receptors, insulin receptor substrates, PI3K/Akt/mTOR and glucose transporter proteins (Liang et al., 2013; Petersen and Shulman, 2018). Insulin resistance (IR) is a pathophysiological phenomenon that refers to the inability of the body’s cells, tissues or body to respond adequately to normal levels of insulin and plays an important role in the development of metabolic syndrome (MS), cardiovascular disease (CVD), non-alcoholic fatty liver disease (NAFLD), and Alzheimer’s disease (AD) (Morris et al., 2014; Sasaki et al., 2020; Oh et al., 2021; PrayGod et al., 2021; Martin-Gonzalez et al., 2022).

**Figure 1:**
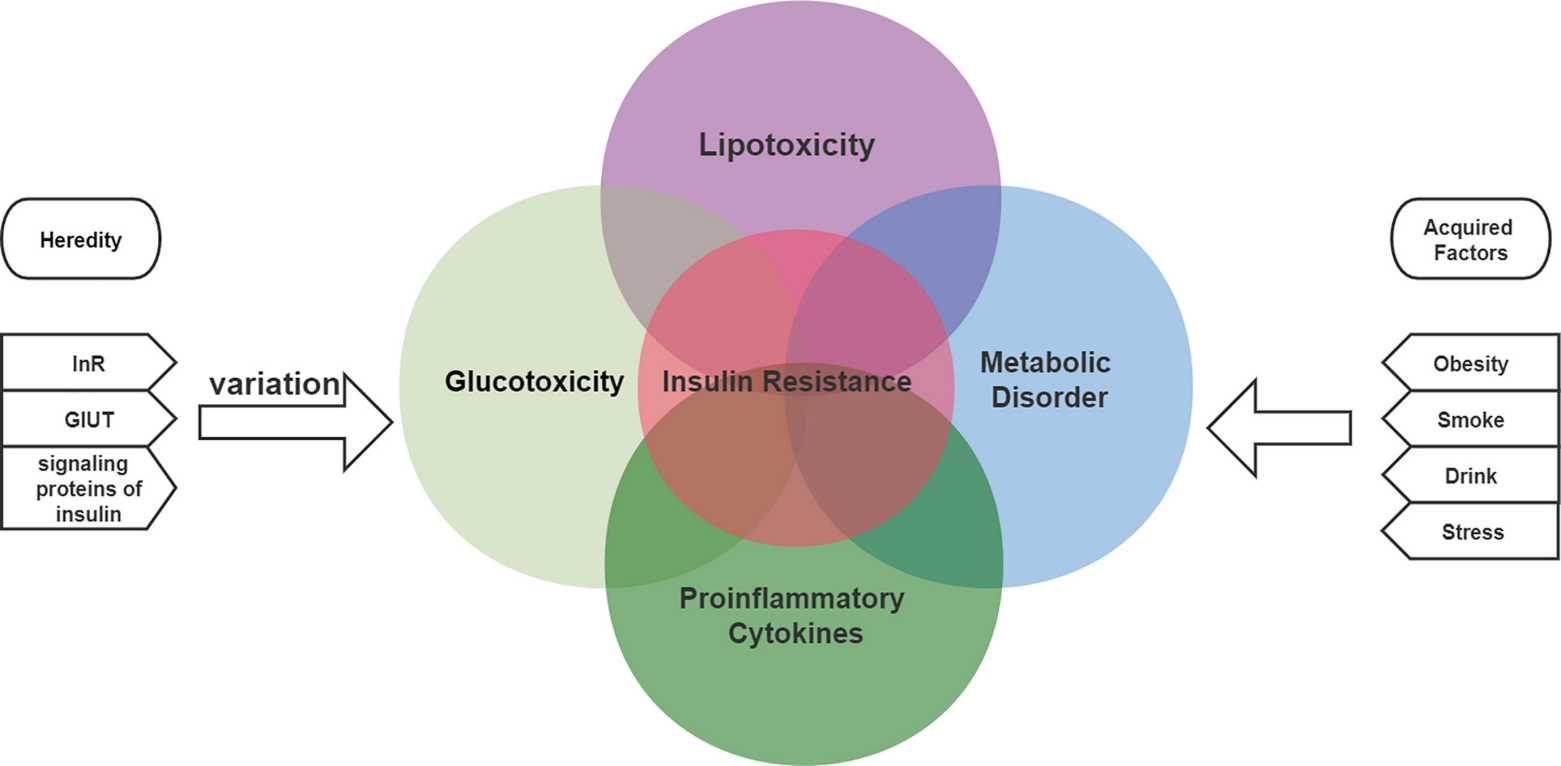
The etiology of insulin resistance includes genetic and acquired factors and is the result of a combination of metabolic disorders, lipotoxicity, glucotoxicity and inflammation.

Insulin resistance may develop through both genetic and acquired factors (Garbossa and Folli, 2017). Common genetic defects include mutations and polymorphisms in insulin receptors, glucose transporters, and signaling proteins involved in insulin signaling. Two of the 17 causes of acquired insulin resistance include obesity, lack of exercise, advanced glycosylation end products (AGE), excess free fatty acids (FFAs), psychological stress, smoking, alcohol intake, or certain drugs (Vlassara and Striker, 2011; Goodpaster and Sparks, 2017). All these factors are associated with a persistent state of low-grade inflammation. Multiple molecular and pathophysiological mechanisms are involved in insulin resistance (Khodabandehloo et al., 2016). Insulin resistance is the result of a combination of metabolic disorders, lipotoxicity, glucotoxicity and inflammation, as shown in **Figure 1** (Khalid et al., 2021).

This review inventories the pathogenicity of *Pseudomonas gingivalis* and insulin resistance by collecting the most recent studies on *Pseudomonas gingivalis* and insulin resistance and discusses the pathological mechanisms associated with insulin resistance. The impact of *Pseudomonas gingivalis* infection in different systems based on insulin resistance is explored, especially the association with diabetes, non-alcoholic fatty liver disease, Alzheimer’s disease, cardiovascular, and skeletal muscle.

Using the Web of science, PubMed, and Google Scholar databases, original research, case series, and review articles published in English through December 2022 were searched for the terms “*Porphyromonas gingivalis*”, “insulin resistance”, and potential related diseases or systemic disorders (i.e., “atherosclerosis,” “diabetes,” “cardiovascular disease,” “neurological disease “, “non-alcoholic fatty liver disease”, “skeletal muscle”). In addition, the reference lists of relevant articles were screened to reduce the risk of missing relevant information and to guide the search for potential associations. The search terms “oral bacteria” and “periodontitis” were also used as substitutes for “*Porphyromonas gingivalis*”, however, only articles that explicitly mentioned this bacterium and reported its potential activity in the organ of interest were retained. However, only articles that explicitly mention this bacterium and report its potential activity in the organ of interest were retained. The main points of interest are: (1) the pathological mechanism of insulin resistance caused by *P. gingivalis*; (2) the association of *P.g* with systemic diseases based on insulin resistance; and (3) treatment.

## 2 Pathogenesis of insulin resistance caused by *P. gingivalis*

### 2.1 Tissue colonization

*P. gingivalis* invades and survives in host tissues by disrupting the gingival epithelial barrier, internalizing into epithelial cells, and enhancing epithelial cell autophagy. Bacteremia may occur after oral surgery and can also lead to systemic transmission of *P. gingivalis*. Disruption of the complement system, degradation of antimicrobial peptides, and disruption of phagocytic function all contribute to the escape of *P. gingivalis*. *P. gingivalis* also suppresses adaptive immunity, allowing *P. gingivalis* to be present in host tissues and persistently cause inflammatory responses. Transit of oral microorganisms to distant organs is thought to play an important role in disease development. Many clinical reports have shown that *P. gingivalis* is the main pathogen of periodontal disease and is often detected in distal organs such as the liver, cardiovascular tissue, cerebrospinal fluid, and tubal-ovarian sites(Iida et al., 2004; Park et al., 2011; Tomas et al., 2012; Yoneda et al., 2012; Ishikawa et al., 2013; Eberhard, 2022).For example, these virulence factors are involved in the pathogenicity of bacteria in infected tissues and make possible their transmission. *In vitro* and clinical studies have shown that *Pseudomonas gingivalis* infiltrates and survives in non-oral human cells (e.g. coronary endothelial cells and placental cells) and may contribute to the local inflammatory response(Champaiboon et al., 2014; Weishan et al., 2016). *Pseudomonas gingivalis* can be detected in many cardiovascular diseases, such as atherosclerosis, myocardial infarction, stroke, aneurysm, pericarditis and pericardial tamponade(Pussinen et al., 2007; Patrakka et al., 2019). *P. gingivalis* DNA was detected in synovial tissue from RA patients, suggesting that the bacterium may be localized intracellularly (Moen et al., 2006; Martinez-Martinez et al., 2009; Ogrendik, 2012; Reichert et al., 2013; Totaro et al., 2013). Carrion, J et al. found that dendritic cells in the blood carry *P. gingivalis* in patients with acute coronary syndrome with chronic periodontitis and can transmit *P. gingivalis* from the oral mucosa to atherosclerotic plaques (Carrion et al., 2012). Furusho, H et al. performed *P. gingivalis* immunohistochemistry in liver biopsy specimens from patients with NASH and found the presence of *P. gingivalis* in the livers of patients with advanced fibrosis NASH(Furusho et al., 2013).

### 2.2 Decreased number and dysfunction of pancreatic islet beta cells

*P. gingivalis* disrupts the local and systemic immune system, causing a systemic inflammatory response that leads to insulin resistance in peripheral insulin-responsive tissues. there is a positive feedback regulatory mechanism between β-cells and insulin-sensitive tissues, where β-cells respond to the demands of liver, skeletal muscle and adipose tissue to increase insulin supply, and over-stimulation of β-cell insulin secretion leads to increased β-cell signaling and oxidative stress, which The resulting hyperinsulinemia leads to β-cell dysfunction and eventually β-cell apoptosis. In the insulin-resistant state, β-cell depletion to maintain normal blood glucose and compensate for insulin requirements is the key to the pathogenesis.

*P. gingivalis* disrupts the local and systemic immune system, causing a systemic inflammatory response that leads to insulin resistance in peripheral insulin-responsive tissues. there is a positive feedback regulatory mechanism between β-cells and insulin-sensitive tissues, where β-cells respond to the demands of liver, skeletal muscle and adipose tissue to increase insulin supply, and over-stimulation of β-cell insulin secretion leads to increased β-cell signaling and oxidative stress, which the resulting hyperinsulinemia leads to β-cell dysfunction and eventually β-cell apoptosis (Cerf, 2013; Wang et al., 2013; Mezza et al., 2019; Sharma et al., 2022). In the insulin-resistant state, β-cell depletion to maintain normal blood glucose and compensate for insulin requirements is the key to the pathogenesis (Ma et al., 2007).

In addition, *P. gingivalis* also migrates to the pancreas, causing inflammation and apoptosis of β-cells. ge Q-M et al. demonstrated that LPS increased the expression of Toll4 receptors on the membrane surface of pancreatic β-cells (Ge et al., 2011). Liza L. Ramenzoni et al. found that *P.g* can induce β-cell inflammation by activating the P13K/AKT signaling pathway through TLR4 to induce pro-inflammatory molecules, while stimulating insulin secretion and increasing β-cell compensatory responses(Ramenzoni et al., 2019). Ilievski, Vladimir et al. found that *P. gingivalis* metastasizes to the pancreas and that beta-cell apoptosis increases, leading to complex changes in islet morphology. serpinE1 appears to be involved in this process (Zhou et al., 2009).

Thus, the production of inflammatory factors, oxidative stress and the activation of signaling cascades associated with cytotoxicity lead to β-cell damage and degradation (Kouidrat et al., 2015; Abedini et al., 2018; Yaribeygi et al., 2019). As the number of beta cells decreases, the condition is further aggravated by the increased insulin requirements of the pancreas to overcome the body’s insulin-resistant state.

### 2.3 Inflammatory reaction

Studies have shown that continuous infusion of Pg can induce chronic systemic inflammation. *P.g*-LPS can be released in local and systemic organs, leading to endotoxemia, triggering regional and systemic inflammation, and promoting insulin resistance (Chiu and Lin, 2008; Baker et al., 2011; Huang et al., 2016).

The inflammatory mechanism by which *P. gingivalis* induces insulin resistance involves the activation of toll-like receptor (TLR) signaling pathway. The innate immune receptors TLR2 and TLR4 are pattern recognition receptors that are expressed in a variety of cell types, including macrophages, hepatocytes, and pancreatic beta cells (Hoshino et al., 1999; Jiang et al., 2000; Iwasaki and Medzhitov, 2004; Genco et al., 2005; Hemmi and Akira, 2005). Toll-like receptors play an important role in the mechanism of inflammation (Nishihara et al., 2009; Blasco-Baque et al., 2017). *P. gingivalis* LPS activates toll-like receptor (TLR)-related signaling pathways, which in turn trigger the release of cytokines and chemokines (Despres et al., 2008; Lee and Seong, 2009; Anhe et al., 2015; Le Sage et al., 2017). A previous study also reported that *P.g*-LPS could promote the production of interleukins and tumor necrosis factors (TNFs) by activating tlr4-related signaling pathways (Fujimoto et al., 2013). Lappin, DF et al. found elevated levels of Toll-like receptor 2 and 4 stimulators in saliva of patients with periodontitis compared to healthy subjects (Lappin et al., 2011). Watanabe, K et al. demonstrated a protective effect of TLR4 mutations on alveolar bone loss and improved glucose homeostasis in mice with periodontitis fed a high-fat diet (Watanabe et al., 2011).

The binding of LPS to TLR4 triggers a signaling cascade that leads to the activation of various pro-inflammatory pathways, such as the nuclear factor kappa B (NF-κB) pathway and the mitogen-activated protein kinase (MAPK) pathway. These pathways can induce the production of pro-inflammatory cytokines, such as tumor necrosis factor-a (TNF-a), interleukin-6,leptin, lipocalin, resistin, and acute phase proteins (Fernandez-Real and Ricart, 1999; Gonzalez et al., 2001; Evans et al., 2002). Advanced glycosylation end products (AGEs) are increased in diabetes and may also lead to inflammation and infection. Several molecules have been shown to induce insulin resistance, such as IL-6, TNF-a, resistin and free fatty acids (Rojo-Botello et al., 2012; Papale et al., 2018; Cattaneo et al., 2019; Lin et al., 2020; Wang et al., 2022b). IL-6 was one of the first cytokines to be considered a predictor or pathological marker of insulin resistance and cardiovascular disease (Mendiola et al., 2017). TNF-α has been shown to promote insulin resistance in different ways, such as TNF-α may increase insulin resistance by inhibiting the entry of glucose into smooth muscle cells (Savage et al., 2007; Rowan et al., 2018; Wang et al., 2022a). There is evidence that anti-tnf antibody treatment improves hepatic IR (Hu et al., 2022; Sinha and Haque, 2022). Resistin, an adipocyte-derived signaling polypeptide thought to have increased expression in inflammatory diseases and diabetes, named for its function in resisting insulin,is also a pro-inflammatory molecule (Hiroshima et al., 2012; Hrishi et al., 2016; Govindaraj et al., 2021). Palmitate, a nutritional free fatty acid (FFA) that activates TLR2, causes inflammation and induces insulin resistance (Hajishengallis, 2014; Ilievski et al., 2017).

**Figure 2:**
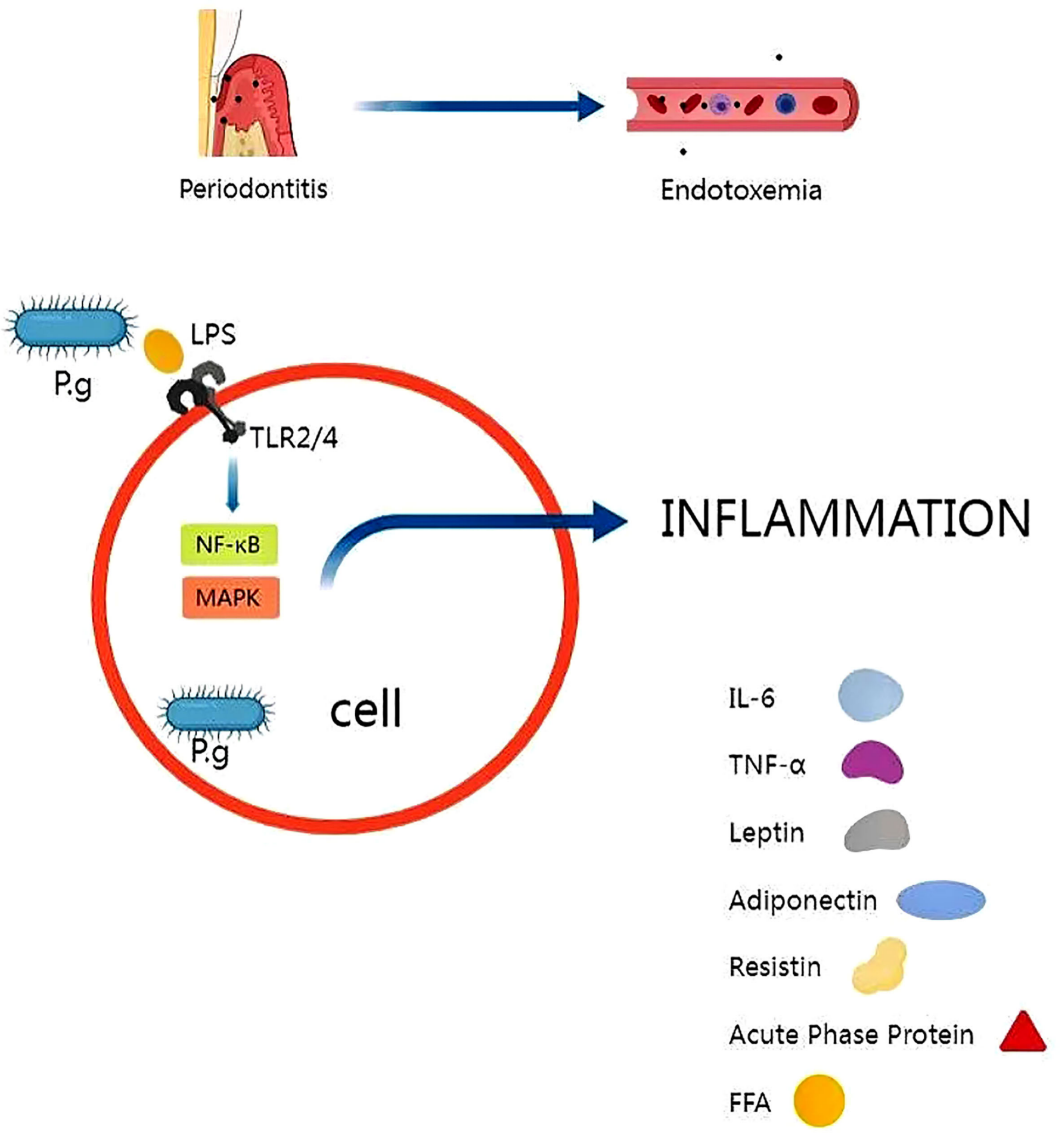
*P.g* is capable of disrupting the immune barrier, causing endotoxemia and triggering regional and systemic inflammation. The release of *P.g*-LPS activates toll-like receptor (TLR)-related signaling pathways, which in turn trigger the release of inflammatory factors. Several molecules have been shown to induce insulin resistance, such as IL-6, TNF-a, resistin and free fatty acids.

Overall, the inflammatory mechanism of insulin resistance induced by *P. gingivalis* involves the activation of TLR signaling pathway and the production of pro-inflammatory cytokines. These findings suggest that periodontal disease may play a role in the development of insulin resistance and type 2 diabetes, and that treating periodontal disease may have beneficial effects on glycemic control in individuals with insulin resistance, as shown in **Figure 2**.

### 2.4 Immune escape

*P. gingivalis* can manipulate the host immune system by producing virulence factors that can interfere with immune cell signaling pathways or by inducing the production of anti-inflammatory cytokines, in order to maintain inflammation, obtain nutrients, and evade host immune killing. e, JZ et al. found that *P. gingivalis* was able to interfere with the immune system and thus induce insulin resistance (He et al., 2022).

The innate and adaptive immune systems are important components of the immune system. *P. gingivalis* interferes with the innate immune response in healthy gums:*P. gingivalis* disrupts the epithelial barrier, inhibits complement-mediated lysis, and achieves intracellular self-proliferation in epithelial cells, which initiates the first step in *Pseudomonas gingivalis* evasion of host immunity (Holmes et al., 2009). Escape of neutrophils and killing by destructive macrophages paves the way for the long-term persistence of *Pseudomonas gingivalis*. Then down-regulates the differentiation and development of adaptive immune cells: The ability to manipulate T-cell differentiation and immune responses provides for the survival of *Pseudomonas gingivalis* in the host (Soto et al., 2022). Low-grade inflammation induced by the innate immune system occurs under the control of the adaptive immune system, and if the immune system is compromised, the presence of bacteria at the site of infection may lead to systemic inflammation, thereby exacerbating insulin resistance (Harding et al., 2017; Ganz et al., 2022).

Huang, X et al. found that in obese individuals, macrophage infiltration and activation in periodontal tissue was significantly downregulated in patients with periodontitis, paralyzing the intrinsic immune response to periodontal disease(2017). Blasco-Baque et al., 2017 found that *P. gingivalis* infection affects antibody- *P.g* production, leading to an impaired specific immune system. P *P. gingivalis*, mediates not only local immune responses within periodontal pockets, but also systemic immune responses that enhance insulin resistance (Cao et al., 2019; Bonomo et al., 2020). He, JZ et al. found that *P. gingivalis* infection leading to dysbiosis of the oral microflora induced an immune response in the spleen of diabetic mice, characterized by a decrease in innate immune cells and an increase in adaptive immune cells, decreasing the levels of IL17-producing monocytes and ILC3 in the spleen, but increasing Th17 cells, which can lead to alveolar bone resorption. This is accompanied by an upregulation of the Th17/Tregs ratio and abnormalities in immune function, while an increase in TH17 cells promotes blocking inflammation and increases the risk of insulin resistance (Greggianin et al., 2023; Xiao et al., 2017).

Insulin plays a key role in the metabolism of immune cells, so insulin resistance caused by *P. gingivalis* infection can further damage the host’s immune function (DiAngelo et al., 2009; Hotamisligil, 2017; van Niekerk et al., 2020). Since glucose is pro-inflammatory, insulin mitigates the deleterious effects of hyperglycemia through metabolic regulation (Sun et al., 2014). In addition, studies in rodents have shown that insulin directly activates the phagocytic and bactericidal activity of immune cells and that islet deficiency can lead to an impaired immune response, which is one of the reasons why diabetic patients are susceptible to co-infections (Yano et al., 2012). Insulin regulates the metabolic reprogramming of T cells, thereby regulating adaptive immunity. *In vitro*, T cells lacking InsR have an attenuated response to antigen, and *in vivo* T cell-specific knockdown of InsR in mice results in reduced antigen-specific immunity to influenza virus infection(Tsai et al., 2018; Shinjyo and Kita, 2021).

### 2.5 Lipotoxicity

Studies have suggested that *P. gingivalis* can contribute to the development of insulin resistance, one proposed mechanism for this effect is through lipotoxicity. Insulin resistance (IR) is associated with lipotoxicity, and chronic inflammation may be a common denominator (Watanabe et al., 2011; Fleetwood et al., 2017; Ni et al., 2018; Pirih et al., 2021; Shaukat et al., 2022; Thouvenot et al., 2022; Yoshimoto et al., 2022). In mice, oral administration of *P. gingivalis* leads to systemic inflammation, increased adiposity and insulin resistance (Artese et al., 2015; Le Sage et al., 2017).

In hepatocytes, *P. gingivalis* can stimulate the production of free fatty acids(FFAs). Several studies have shown that FFAs can synergize with LPS to enhance the inflammatory response.TLR2 is an important receptor for macrophage recognition of *P. gingivalis* and mediates the destructive chronic inflammatory response(Nishimura et al., 2015; Marques et al., 2022). It was found that TLR2 can be activated by palmitate, a free fatty acid (FFA), leading to inflammation and induction of insulin resistance (Palmer et al., 2012; Furusho et al., 2013). lu, Z et al. found that FFAs can increase the expression of lps-triggered inflammatory molecules via CD36, leading to strong CD36-mediated inflammatory signaling and an intrinsic cellular immune response (Lu et al., 2017). In adipocytes, *P. gingivalis* can induce inflammation and the release of pro-inflammatory cytokines, which can also contribute to insulin resistance.

**Figure 3:**
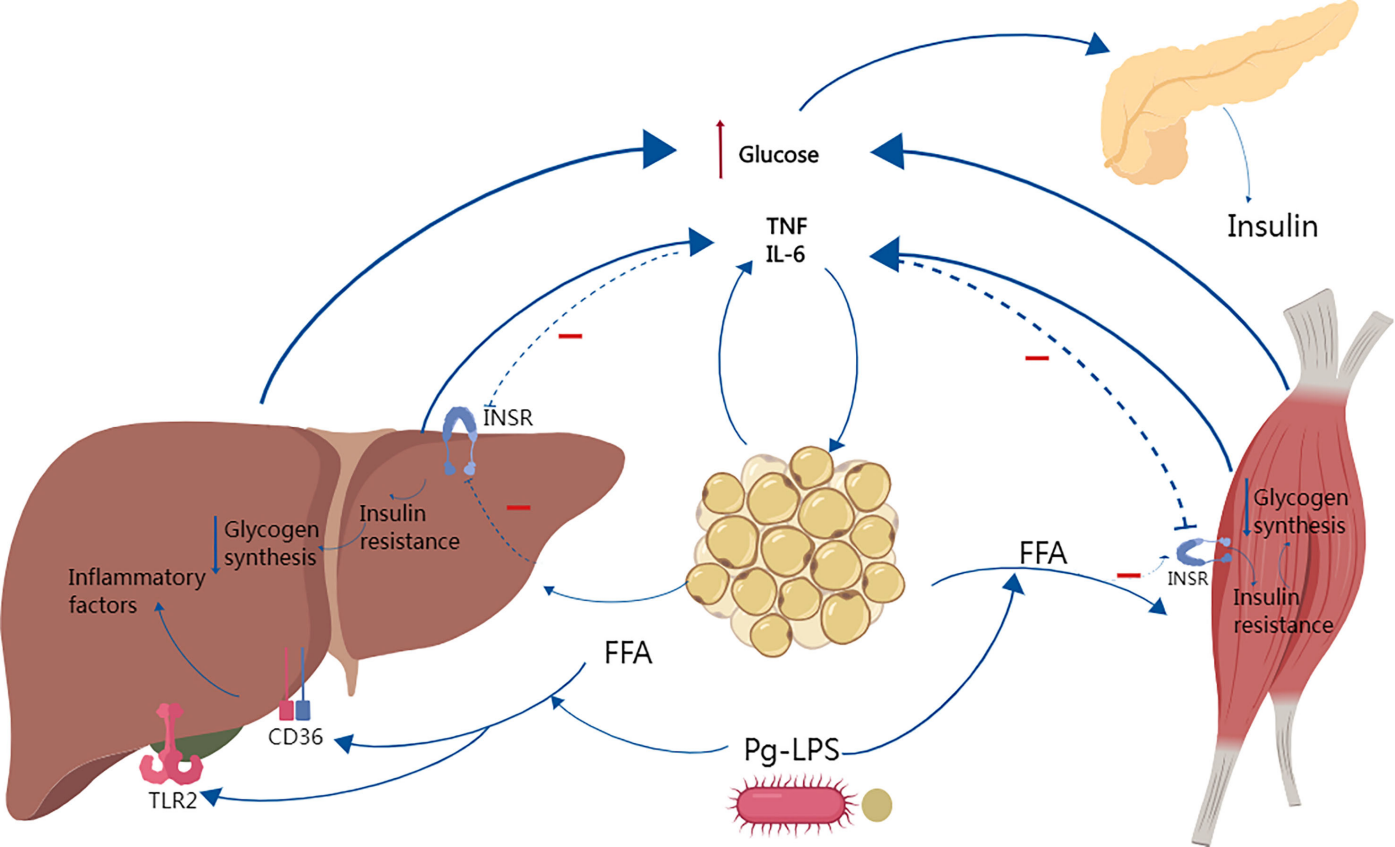
Studies have shown that *Porphyromonas gingivalis* causes systemic inflammation, increased adiposity and insulin resistance. In obese individuals, increased free fatty acid FFAs decrease insulin sensitivity by inhibiting insulin-mediated glucose uptake and reduce glycogen synthesis, and elevated circulating glucose increases insulin secretion, leading to hyperinsulinemia. Several studies have shown that FFAs can synergize with P.g-LPS to activate TLR2 and CD36, enhancing the inflammatory response and increasing the secretion of inflammatory factors such as IL-6 and TNF-α, which in turn further aggravate insulin resistance.

Excess free fatty acids (FFAs) in the body can form ectopic fat accumulation, and obesity or ectopic fat accumulation induces an innate immune response, followed by immune cell aggregation, which can interfere with insulin signaling and promote insulin resistance, as shown in **Figure 3** (Peng et al., 2011; Shikama, 2018; Halade and Tourki, 2019; Jung et al., 2022).

### 2.6 Dysbiosis of the gut flora

The gut microbiota is part of the host’s metabolic system and actively regulates energy balance (Wang et al., 2017). Dysbiosis of the gut flora is associated with a number of chronic inflammatory diseases such as diabetes and neurodegenerative diseases, including AD (Guo et al., 2017; Chen et al., 2021; Leblhuber et al., 2021; Rodriguez and Delzenne, 2021; Romanenko et al., 2021; Zawada et al., 2021).

There is some evidence to suggest that *P. gingivalis* can indirectly contribute to insulin resistance by altering the composition of the gut microbiome. Changes in the gut microbiome can lead to dysbiosis, which can promote inflammation and insulin resistance.

It was found that the microbial network of the intestinal microbiota changed significantly after *P. gingivalis* administration. Kashiwagi et al., 2021 found that oral administration of *P. gingivalis* caused changes in the intestinal flora and led to disturbances in entero-hepatic metabolism, which exacerbated hyperglycemia in an obese type 2 diabetic mouse model (Kashiwagi et al., 2021). In a 2014 study, Arimatsu, K et al. found significant changes in the intestinal microbiota of mice following oral administration of *P. gingivalis* a typical periodontal pathogen, with a significant increase in populations belonging to the family Bacillariophyceae, consistent with an increase in insulin resistance and systemic inflammation. The findings suggest that *P. gingivalis* can induce insulin resistance by altering the intestinal flora (Arimatsu et al., 2014a).

In obese individuals, *P. gingivalis* periodontal pathogens or E. coli LPS increase the production of pro-inflammatory factors,promote the downregulation of reactive oxygen species (ROS) and antioxidant defense systems, lead to oxidative stress, and trigger insulin resistance (de Faria and Fortunato, 2020; Zhou et al., 2021). In addition, the native flora plays a key role in host defense against infection by stimulating mucosal immune defenses (e.g., antimicrobial peptide and IgA release) and limiting invading microbes (Astapova and Leff, 2012; Lv et al., 2019). Thus, dysbiosis is associated with susceptibility to infection, which may further accelerate immunometabolic imbalance (Lazar et al., 2018).

## 3 Association of *P. gingivalis* with systemic diseases based on insulin resistance

### 3.1 Diabetes

Type 2 diabetes is a metabolic disease that is a major public health problem worldwide. The bidirectional association between periodontitis and diabetes has been widely accepted (Lalla and Papapanou, 2011).Results of cross-sectional studies have shown that patients with periodontitis have a higher incidence of insulin resistance (IR) (Bhat and Watanabe, 2015). Insulin resistance is an important cause of type 2 diabetes mellitus (T2DM) (Tian et al., 2020). *P. gingivalis* is the main causative agent of periodontitis, and several studies have shown that *P. gingivalis* can cause insulin resistance, which can lead to diabetes.

Ilievski, V et al. found in a 2020 study that oral administration of the periodontal pathogen *P. gingivalis* in mice led to insulin resistance, hyperinsulinemia and glucose intolerance. Sasaki, N et al. showed that in mice fed a high-fat diet, intravenous *P. gingivalis* led to impaired glucose tolerance, insulin resistance and hepatic steatosis (Sasaki et al., 2018). Ilievski, Vladimir et al. found that oral administration of *P. gingivalis* induced prediabetes (Ilievski et al., 2016). Seyama et al., 2020 study found that outer membrane vesicles released by *P. gingivalis* attenuated Akt/glycogen synthase kinase-3β (GSK-3β) signaling in hepatic HepG2 cells, inducing insulin resistance and allowing insulin-induced reduction in hepatic glycogen synthesis, thereby maintaining high blood glucose levels(Seyama et al., 2020). Bhat et al., 2014 demonstrated that *P.g*-LPS stimulates insulin secretion from the pancreatic β-cell lineage MIN cells.*P.g*-LPS may be important in the development of b-cell compensation and insulin resistance in patients with periodontitis in prediabetes (Bhat et al., 2014).Tian, J et al. an animal study showed that *P. gingivalis* periodontal infection significantly upregulated plasma branched-chain amino acid levels and exacerbated The plasma branched-chain amino acid biosynthetic pathway may provide a potential target for the link between periodontitis and T2DM (Kang et al., 2022).

**Figure 4:**
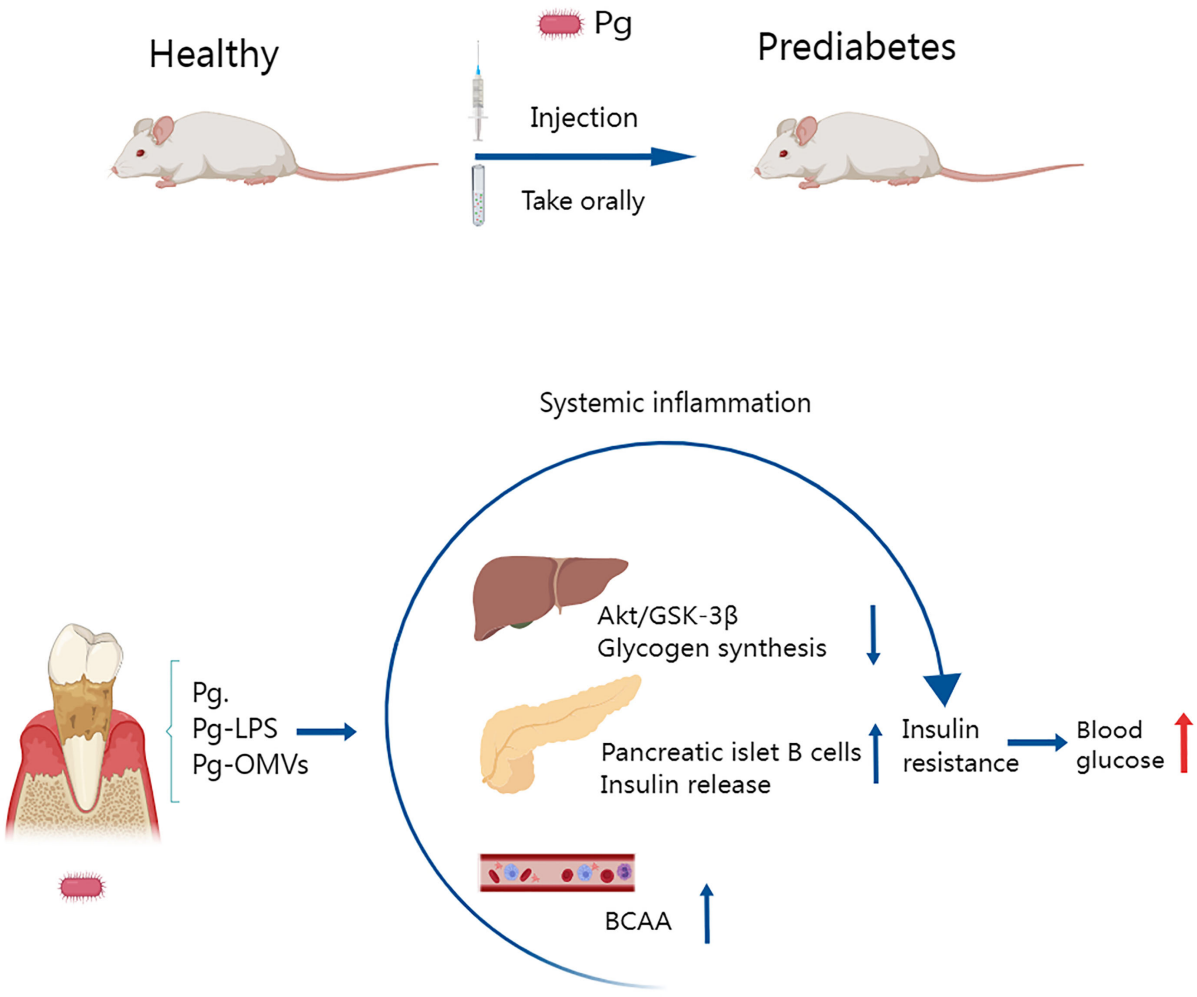
Several studies have shown that oral administration or injection of *Porphyromonas gingivalis* in mice leads to prediabetes and that insulin resistance is an important pathological mechanism involved. *Porphyromonas gingivalis* and the vesicles and LPS it releases disrupt hepatic glycogen synthase, cause pancreatic β-cell compensation, and elevate plasma branched-chain amino acids, which lead to decreased insulin function and reduced synthesis, inducing insulin resistance and leading to elevated blood glucose.

Individuals with type 2 diabetes and periodontitis are severely impaired by the ability of one disease to exacerbate the other, and disease-associated inflammation is thought to be one mechanism that fuels this pathogenic cycle (Zhu et al., 2014). *P. gingivalis* can induce a sustained elevation of inflammatory factors associated with type 2 diabetes. Bacterial endotoxin/lipopolysaccharide (LPS) from infected periodontal sites readily invades the circulatory system and induces endotoxemia (Bhat et al., 2014). Thus, bacterial endotoxins may mediate inflammatory responses in distant organs, leading to increased levels of systemic inflammatory mediators, which exacerbate insulin resistance and promote the development of diabetes, as shown in **Figure 4** (Ramenzoni et al., 2019).

### 3.2 Non-alcoholic fatty liver disease

Non-alcoholic fatty liver disease (NAFLD) is a metabolic stress liver injury disease closely related to insulin resistance and genetic susceptibility. It is characterized by excessive invasion of triglycerides in hepatocytes independent of alcohol intake. Multiple risk factors are thought to contribute to the pathogenesis of this disease, including metabolic disorders, immune response(Thalla et al., 2022). Periodontitis is considered a risk factor for metabolic disorders, and periodontal disease bacteria can also exacerbate NAFLD (Nagao et al., 2021). *Porphyromonas gingivalis*-induced endotoxemia exacerbates NAFLD, increases insulin resistance, and inhibits glucose metabolism (Ezhilarasan, 2021).Studies in mice have shown that P. gingivalis can be localized in the autophagosomes and lysosomes of HepG2 cells (Souccar et al., 2010). Furusho, H et al. performed P. gingivalis immunohistochemistry in liver biopsy specimens from NAFLD patients and detected P. gingivalis in Kupffer cells and hepatocytes and found that P. gingivalis infection may upregulate the P.g-LPS-TLR2 pathway and activation of the inflammasome play an important role in the progression of NAFLD. P.g-LPS may also induce activation of the NF-κB signaling pathway, which plays a crucial role in inflammation and is associated with the development of obesity-induced insulin resistance, metabolic syndrome, and NAFLD (Nakahara et al., 2018).

Chronic low-grade inflammation induced by *P. gingivalis* infection induces insulin resistance, which affects hepatic glucolipid metabolism and consequently leads to lipid accumulation in hepatocytes. In addition to this, a study by Ahn et al., 2021 found that *P. gingivalis* induced the progression of nonalcoholic fatty liver disease in high-fat-fed mice by upregulating the CD36-PPARγ axis (Ahn et al., 2021). As one of the important metabolic enzymes in fatty acids, fatty acid translocase 36 (CD36) is widely expressed in various cells (myocytes, monocytes, macrophages, hepatocytes) (Thanakun et al., 2014). It not only mediates the uptake and transport of fatty acids directly, but also recognizes many endogenous metabolites of inflammation(Miquilena-Colina et al., 2011). Chronic low-grade inflammation caused by *P. gingivalis* has been reported to upregulate CD36 expression in hepatocytes and increase hepatic fat accumulation, which in turn leads to nonalcoholic fatty liver disease(Ni et al., 2018).

Recently, a growing body of evidence supports the association between NAFLD and dysbiosis of the oral and gut microbiota(Arimatsu et al., 2014a). Oral administration or injection of *Porphyromonas gingivalis* to mice has been reported to induce dysbiosis of the intestinal flora and promote the development of NAFLD with insulin resistance and systemic chronic inflammation(Bajaj et al., 2017). A significant increase in the abundance of *Allobaculum* spp. was found in mice receiving *Porphyromonas gingivalis*, which coincided with an increase in insulin resistance and systemic inflammation (Lee et al., 2015). However, *Lactobacillus reuteri* was reduced, which decreased insulin sensitivity (Morello et al., 2018).

### 3.3 Alzheimer’s disease

Alzheimer’s disease (AD), the most ordinary form of dementia, is a neurodegenerative disease characterized by cognitive decline due to the accumulation of β-amyloid (a β) plaques and neurofibrillary tangles in the brain.

Results from cross-sectional and longitudinal studies suggest that periodontitis is strongly associated with cognitive impairment (CI) and Alzheimer’s disease (AD).Stein, PS et al. found that periodontal disease may promote the onset/progression of AD and that *P. gingivalis* serum antibody levels are increased in patients with Alzheimer’s disease (AD) (Stein et al., 2012). Duan et al. 2022a study found that periodontitis may be a risk factor for exacerbation of cognitive dysfunction in patients with AD-like neurodegeneration, possibly through impairment of insulin signaling pathways, stimulation of glial proliferation and neuroinflammation(Duan et al., 2022a).

*P. gingivalis* is a gram-negative bacterium found in the oral cavity that causes periodontal disease. There is evidence of a strong association between *P. gingivalis* infection and Alzheimer’s disease AD, and it has been found in the brains of AD patients (Dominy et al., 2019; Bahar et al., 2021). Pseudomonas gingivalis infection can trigger inflammation of the peripheral and central nervous system in affected individuals, leading to cognitive decline. In mice, oral *P. gingivalis* infection leads to brain colonization and ad-like pathogenesis, including complement activation and β-amyloid formation, suggesting a potential mechanistic link between periodontal disease and AD (Poole et al., 2015). A systematic review of preclinical studies also found that *P. gingivalis* infection induces an inflammatory response and tissue degeneration in the brain, which is associated with cognitive impairment (Fernandes Costa et al., 2021). A 2018 study by Ilievski, V et al. found neurodegeneration and extracellular Aβ formation in young adult WT mice after repeated oral *P. gingivalis* administration. The neuropathological features observed in this study strongly suggest that low-grade chronic periodontal pathogen infection can lead to neuropathological development consistent with AD (Ilievski et al., 2018).

Several studies have shown that systemic infections caused by *Porphyromonas gingivalis* may affect the inflammatory state of the central nervous system (Fung et al., 2012). Inflammation is a major driver of insulin resistance and defective insulin signaling cascades. Dysregulation of brain IR and brain insulin signaling may play a key role in the pathogenesis of AD (Duan et al., 2022a). Bahar et al., 2021 study found neuroinflammation in the form of reactive microglia and astrocytes in *P. gingivalis* W83-infected db/db mice, and that key genes in the insulin signaling pathway (INSR, IGF1, IRS, IDE, PIK3R, SGK1, GYS, GSK3B, AKT1) mRNA abundance were upregulated, suggesting that *P. gingivalis* oral infection may exacerbate insulin resistance in the brain of db/db mice (Bahar et al., 2021). Insulin plays a key role in the regulation of immunometabolism in the central nervous system and periphery, and dysfunction of these signaling pathways has been associated with cognitive impairment.

### 3.4 Cardiovascular disease

Cardiovascular disease is the most common cause of death in industrialized countries. in 2014, cardiovascular disease accounted for 23.4% of deaths in the United States. Clinical and experimental findings suggest a strong link between periodontal disease and atherosclerosis (Wallet et al., 2018).

Kuroe et al., 2004 found that *P. gingivalis* infection was associated with atherosclerosis in non-obese Japanese patients with type 2 diabetes mellitus (Kuroe et al., 2004).Studies have shown that *P. gingivalis* infection accelerates atherosclerosis in hyperlipidemic animals and humans (Ikai et al., 2015; Nagaoka et al., 2017; Nakayama and Ohara, 2017; Naginyte et al., 2019). *P. gingivalis* and other oral bacteria are often detected in human atherosclerotic plaques and have been cultured from them(Manning-Tobin et al., 2009; Masters et al., 2010). Transient oral bacteremia occurs after activities such as routine dental treatment or daily oral hygiene (Mathur et al., 2018). This route of entry into the vascular system can serve as a direct pathway for bacteria to enter the endothelium (Mayer et al., 2017).Alternatively, oral bacteria may enter the vessel wall indirectly due to transport of intracellular bacteria by host immune cells to sites of atherosclerotic plaque formation or due to persistent inflammation in the oral cavity affecting the blood vessels. At the cellular level, *P. gingivalis* can invade human vascular cells, including human umbilical vein endothelial cells and coronary cells, and induce a range of cytokines and cell adhesion molecules that are characteristic of activated endothelial cells in atherosclerosis (Mehta et al., 2007; Mizoguchi et al., 2007).

*P.g* infection activates inflammatory pathways and increases the release of inflammatory factors, which is thought to be a novel mechanism by which periodontitis increases the risk of cardiovascular disease and insulin resistance (Lappin et al., 2011). Lappin, DF et al. found that experimental administration of TLR2 or TLR4 stimulants to mice resulted in insulin resistance as well as significantly accelerated atherosclerosis, and that deletion of the TLR2 or TLR4 genes prevented the development of these diseases. (Ioana et al., 2012).

Almudena Gómez-Hernández et al. found that severe hepatic insulin resistance is sufficient to cause cardiovascular insulin resistance and dysfunction. The different manifestations of insulin resistance, including dyslipidemia, hyperglycemia, inflammation, and obesity, may be mediators of insulin resistance co-producing endothelial dysfunction. Thus, hyperlipidemia, hyperglycemia and pro-inflammatory cytokines are known to selectively impair the PI3K/AKT/eNOS pathway, increase oxidative stress, and enhance ET-1 release via the intact or enhanced MAPK-ET1 pathway in endothelial cells. Insulin resistance states are associated with metabolic abnormalities, including glucotoxicity, lipotoxicity and inflammation, which can also lead to endothelial dysfunction(Gomez-Hernandez et al., 2021).

### 3.5 Skeletal muscle

Skeletal muscle is the most representative tissue of the body and is necessary for voluntary movement and body position (Baker et al., 2011). In addition, skeletal muscle plays an important role in nutritional homeostasis, thermoregulation, endocrine system regulation, energy metabolism and glucose uptake (Batsis and Villareal, 2018).About a quarter of the ingested glucose is stored in skeletal muscle as glycogen and used as a source of energy (Bolyen et al., 2019). The clinical consequences of insulin resistance and compensatory hyperinsulinemia have become a major public health problem. Myasthenia gravis is a progressive reduction in muscle mass with age. Sarcopenic obesity is characterized by an increase in body fat mass and a decrease in muscle mass (Zhu et al., 2013). The prevalence of skeletal muscle obesity is expected to increase as the elderly population increases (Zimowska et al., 2017).

*Porphyromonas gingivalis* infection leads to an increase in systemic inflammatory mediators, including TNF-α, and causes insulin resistance (Greggianin et al., 2023). Watanabe, K et al. found that *P. gingivalis* infection caused sarcopenic obesity and metabolic dysfunction associated with floundering muscle. The findings suggest that prevention and treatment of periodontal disease may contribute to the incidence of muscle-reducing obesity. Many studies have shown that periodontitis affects the diabetic state and *P. gingivalis* can increase insulin resistance by inhibiting glucose entry into smooth muscle cells using LPS and TNF-α (Sakalauskiene et al., 2014).

The findings suggest that *P. gingivalis* infection can trigger insulin resistance, a risk factor for metabolic syndrome and skeletal muscle metabolic dysfunction, one pathway of which is alterations in the gut microbiota. In a study by Watanabe et al., 2021, they found significant changes in the microbial network of the gut microbiota in mice taking *P. gingivalis*, compared to control flounder muscles that exhibited fat infiltration and low glucose uptake, higher TNF-α expression and lower insulin signaling, and TNF-α reduced glucose uptake in C2C12 myogenic cells *in vitro*,are explained in detail in **Table 1**. (Chandra and Shashikumarr, 2019; Watanabe et al., 2021).

**Table 1:** Association of *Porphyromonas gingivalis* with systemic diseases based on insulin resistance.

Disease	Research evidence	Proposed direction of causality and or mechanism	Reference
Diabetes	• Oral administration of *P.g* in mouse leads to insulin resistance • Intravenous *P.g* leads to impaired glucose tolerance, insulin resistance and hepatic steatosis •*P.g*-OMVs can attenuate Akt/GSK-3β signaling in hepatocytes • *P.g*-LPS stimulates pancreas β Cell line MIN6 secrete insulin •The level of AAA was significantly up-regulated by infection of *P.g*	*P.g* causes systemic inflammation, reduces glycogen synthesis, and induces β-Cell compensation, leading to pre diabetes	(Lalla and Papapanou, 2011) (Bhat and Watanabe, 2015) (Tian et al., 2020) (Sasaki et al., 2018) (Ilievski et al., 2016) (Seyama et al., 2020) (Bhat et al., 2014) (Kang et al., 2022) (Zhu et al., 2014) (Bhat et al., 2014) (Ramenzoni et al., 2019)
Nonalcoholic fatty liver disease(NAFLD)	• *P.g* was detected in Kupffer cells and hepatocytes of NAFLD patients • *P.g* infection may up-regulate the *P.g-* LPS-TLR2 pathway and activate inflammatory enzymes • *P.g* can up-regulate CD36-PPAR γ axis	Chronic low-grade inflammation caused by *P.g* infection induces insulin resistance, which affects hepatic glucolipid metabolism and then leads to NAFLD	(Thalla et al., 2022) (Nagao et al., 2021) (Yoneda et al., 2012) (Souccar et al., 2010) (Ahn et al., 2021) (Thanakun et al., 2014) (Miquilena-Colina et al., 2011) (Ni et al., 2018)
Alzheimer’s disease	• Increased serum antibody level of *P.g* in patients with AD • *P.g* infected db/db mice, and neuroinflammation occurred in the form of reactive microglia and astrocytes • Upregulation of mRNA abundance of key genes in insulin signaling pathway	*P.g* infection causes insulin resistance in the nervous system, further aggravates the inflammatory reaction and leads to neurodegeneration	(Stein et al., 2012) (Duan et al., 2022a) (Bahar et al., 2021) (Dominy et al., 2019) (Poole et al., 2015) (Fernandes Costa et al., 2021) (Ilievski et al., 2018) (Bahar et al., 2021)
Cardiovascular disease	• P.g is often detected in human atherosclerotic plaque. • TLR2 or TLR4 stimulators can cause insulin resistance and significantly accelerate atherosclerosis. • Severe liver insulin resistance is enough to cause cardiovascular insulin resistance and dysfunction	*P.g* infection causes insulin resistance, leads to metabolic abnormalities, and aggravates endothelial dysfunction	(Wallet et al., 2018) (Kuroe et al., 2004) (Monasterio et al., 2019) (Nagano et al., 2017) (Naginyte et al., 2019) (Nagaoka et al., 2017) (Nakayama and Ohara, 2017) (Manning-Tobin et al., 2009) (Masters et al., 2010) (Mathur et al., 2018) (Mayer et al., 2017) (Mehta et al., 2007) (Mizoguchi et al., 2007) (Ioana et al., 2012) (Gomez-Hernandez et al., 2021)
Sarcopenia	• *P.g* infection causes myopenic obesity of soleus muscle and is associated with metabolic dysfunction. • The soleus muscle of *P.g*-infected mice showed fat infiltration and low glucose uptake. • *P.g* can utilize LPS and TNF- α Inhibit glucose entry into smooth muscle cells and increase insulin resistance	*P.g* infection can cause inflammatory infiltration of skeletal muscle, induce insulin resistance and lead to skeletal muscle metabolic dysfunction	(Baker et al., 2011) (Batsis and Villareal, 2018) (Bolyen et al., 2019) (Zhu et al., 2013) (Zimowska et al., 2017) (Sakalauskiene et al., 2014) (Watanabe et al., 2021)

## 4 Therapy

### 4.1 Periodontal treatment

Ni et al., 2018 study showed that periodontitis increases insulin resistance and that scaling and root planning improves insulin resistance (Ni et al., 2018). Laser treatment is an effective method to improve clinical and microbiological parameters in patients with diabetes mellitus with chronic pancreatitis. In addition, there is a better improvement in glycemic control. Therefore, patients with delayed wound healing, like DM2 with CP, can be effectively treated with laser as an adjunct to non-surgical periodontal treatment for better outcomes (Chandra and Shashikumarr, 2019).

### 4.2 Plant polyphenols

Polyphenol therapies have attracted a lot of attention in helping to counteract the harmful effects of periodontal bacteria and improve insulin resistance (Thouvenot et al., 2022). During periodontal disease, polyphenols exert antimicrobial effects and modulate the host inflammatory response. In addition, their anti-inflammatory activity may help improve adipose tissue function and insulin sensitivity during obesity (Gao et al., 2014). Notably, polyphenols are considered to be the most abundant antioxidants in fruits, vegetables and beverages of plant origin (Gurav and Jadhav, 2011; Furugen et al., 2013; Everard et al., 2014). Le Sage, F et al. demonstrated that polyphenols extracted from the medicinal plant Amaranthus longifolia (Antirhea borbonica), mentioned in the French Pharmacopoeia, were used for their antidiabetic effects and protected preadipocytes from pro-inflammatory agents (e.g. E. coli LPS) (Bedard and Krause, 2007). Plant polyphenols reduced the pro-inflammatory effect of LPS. At the same time, polyphenols increased the production of lipocalin and PPARγ, which are known to be key anti-inflammatory and insulin-sensitizing mediators, and to some extent reversed the oxidative stress effects on cells.

### 4.3 Immunotherapy

*P.g* - LPS is responsible for the metabolic damage caused by periodontitis, and it is important to reduce insulin resistance and type 2 diabetes through anti-inflammatory strategies that target the local immune system. *P.g*-lps-based suppressive therapies and antibiotics that directly target Porphyromonas can prevent the deleterious effects of periodontitis on glucose homeostasis in diabetic patients. In addition, vaccination against *P.g* reduces the effect of periodontitis on glucose metabolism. It was found that treatment with inactivated *P.g* prior to periodontal infection induced specific antibodies against *P.g* and protected mice from the metabolic impairment caused by periodontitis (Satoh and Ishihara, 2020). In addition, it was found that anti-TNF-a IgG inhibited the expression of TNF-a mRNA and IL-6 mRNA in the liver of diabetic mice after inoculation with *P. gingivalis*, are explained in detail in **Table 2** (Nishihara et al., 2009).

**Table 2:** Treatment methods related to *P.gingivalis*.

Category	Therapeutic measures	Function	Reference
Periodontal initial therapy	scaling 、root planning、Laser Treatment	Improves insulin resistance and Blood glucose control, promotes wound healing	(Ni et al., 2018) (Chandra and Shashikumarr, 2019)
Medication	Plant polyphenol	Reduce the proinflammatory effect of LPS and increase insulin sensitivity	(Thouvenot et al., 2022) (Gao et al., 2014) (Furugen et al., 2013) (Everard et al., 2014) (Gurav and Jadhav, 2011) (Bedard and Krause, 2007)
Immunotherapy	*P.g* vaccine	Reduce inflammatory factor TNF- α And IL-6 expression	(Satoh and Ishihara, 2020) (Nishihara et al., 2009)

## 5 Conclusion

*P.g* is the main causative agent of periodontitis, and *P.g* infection causes local and systemic inflammation while disrupting the immune system and further aggravating the inflammatory response, thereby inducing insulin resistance. In addition, *P.g* infection leads to β-cell dysfunction with reduced number, which makes insulin resistance more severe. *P.g* infection leads to intestinal flora disorder, which disrupts local immune defense and releases both *P.g* and LPS into blood. Based on insulin resistance, *P.g* can cause systemic diseases such as diabetes, non-alcoholic fatty liver disease, cardiovascular disease, Alzheimer’s disease, and sarcopenia. Based on this theory, the basic treatment of periodontal disease and the systemic therapy for P.g have received attention and become the hot topic nowadays. The association of *Porphyromonas gingivalis* with systemic diseases has been demonstrated, with insulin resistance being an important pathological mechanism, but the associated molecular mechanisms have not been elucidated and require further experimental confirmation. The effect of *Porphyromonas gingivalis* on skeletal muscle metabolism is a relatively weak part of the picture. In addition, the effect of *Porphyromonas gingivalis* on immune cells and how it further induces insulin resistance is a part that needs to be further explored by basic research.

## Author contributions

SJ wrote the manuscript and prepared figures. XL and QD participated in the design, revision, and final approval of the manuscript. All authors contributed to the article and approved the submitted version.
